# Influence of Rural Social Capital and Production Mode on the Subjective Well-Being of Farmers and Herdsmen: Empirical Discovery on Farmers and Herdsmen in Inner Mongolia

**DOI:** 10.3390/ijerph19020695

**Published:** 2022-01-08

**Authors:** Wenlong Li, Suocheng Dong, Haiying Lin, Yu Li, Zehong Li, Zhuang Jin, Bing Xia

**Affiliations:** 1 College of Resources and Environmental Economics, Inner Mongolia University of Finance and Economics, Hohhot 010070, China; nmgliwenlong@126.com; 2 Institute of Geographic Sciences and Natural Resources Research, Chinese Academy of Sciences, Beijing 100101, China; liy@igsnrr.ac.cn (Y.L.); lizehong@igsnrr.ac.cn (Z.L.); 3 College of Tourism, Henan Normal Universality, Xinxiang 453007, China; 4 College of Resources and Environment, University of Chinese Academy of Sciences, Beijing 100049, China; 5 College of Business, Inner Mongolia University of Finance and Economics, Hohhot 010070, China; linhaiying0912@163.com (H.L.); jinzhuang@sina.com (Z.J.); 6 College of Economics and Management, Baotou Teacher’s College, Baotou 014030, China

**Keywords:** rural social capital, production mode, subjective well-being of farmers and herdsmen, agropastoral transition zone (APTZ), sustainable development goals (SDGs), Inner Mongolia

## Abstract

Rural areas are crucial to the realization of sustainable development goals (SDGs). Rural social capital is indispensable for these areas to fulfil the SDGs. As China pursues rural revitalization, it is essential to achieve the sustainable development of rural areas within the agropastoral transition zone (APTZ) in northern China. The same applies to the SDGs’ realization in other APTZs across the globe. From the micro perspective of individual farmers and herdsmen, this article collected 732 microscopic datapoints through repeated rural surveys, and adopted the multivariate ordered probit model to empirically analyze how the subjective well-being of farmers and herdsmen in northern China’s APTZ was affected by the individual (person) and collective (community) layers of rural social capital. Specifically, the production mode was introduced to study the relationship between social capital and subjective well-being, and social capital was measured by a self-designed theoretical analysis framework, which covered six dimensions and two layers (person and community). It was verified that the individual social capital and collective social capital were mutually replaceable in terms of the effect on the subjective well-being of farmers and herdsmen. Additionally, this article examined the influence of different production modes on the social capital and subjective well-being of farmers and herdsmen, and discussed how the same amount of social capital contributes differently to the subjective well-being of farmers and herdsmen under different production models. The results showed that: (1) Social capital significantly promoted the subjective well-being of farmers and herdsmen, and social network was the leading contributor among the dimensions of individual social capital, while social trust was the leading contributor among the dimensions of collective social capital. By the contribution to the subjective well-being of farmers and herdsmen, the six dimensions of social capital can be ranked as social network > social trust > social participation > social standard > social fame > common vision. (2) Individual social capital and collective social capital were mutually replaceable in terms of enhancing the subjective well-being of farmers and herdsmen; when the individual social capital was insufficient, the collective social capital would exert a much greater influence on the subjective well-being of farmers and herdsmen; when the individual social capital grows, the farmers and herdsmen would depend less on collective social capital. (3) The same amount of social capital contributes differently to the subjective well-being of farmers and herdsmen under different production models; by contribution strength, the production models can be ranked as pure herdsmen (PH) > pure farmers (PF) > non-farmers/herdsmen (NFH) > farmers + herdsmen (FH). Finally, several policy suggestions were provided to improve the subjective well-being of farmers and herdsmen in APTZ. The results show that collective social capital eliminates the negative effect of individual social capital shortage on the subjective well-being of farmers and herdsmen. Thus, it is suggested to consider not only the construction of macroscopic hardware environment, but also to roll out policies and measures to improve the subjective well-being from the micro perspective of farmers and herdsmen. These suggestions are concentrated on the implementation of China’s rural revitalization strategy, and the creation of relevant institutions and cultural environments, as well as the optimization of the internal hierarchy of farmers and herdsmen’s social capital. The research results may help to promote the subjective well-being of farmers and herdsmen in rural areas within the APTZs of China and the world, and provide a reference and a path to realize SDGs in China and similar places across the globe.

## 1. Introduction

Rural development has been an increasingly prominent problem for most countries, considering the background of global industrialization and urbanization [1,2]. The sustainable development of rural areas is important to the realization of several sustainable development goals (SDGs), namely, end poverty in all its forms everywhere (SDG1); promote sustained, inclusive, and sustainable economic growth, full and productive employment, and decent work for all (SDG8); reduce inequality within and among countries (SGD10); make cities and human settlements inclusive, safe, resilient, and sustainable (SGD11) [3]. It is now a scientific and realistic problem that must be directly addressed by farmers, researchers, and decision makers [4]. The improvement of rural residents’ subjective well-being is a core proposition and important purpose of sustainable rural development, a key to the realization of SDGs in rural areas across the globe, and a hot topic among researchers in sustainable development [5,6]. The growing social capital helps to weave a healthy network among individuals, improve individual well-being through community attachment, trust, and safety, and promote coordinated development between regions [5,6]. Therefore, social capital is widely regarded as a necessary and sufficient condition for the pursuit of sustainable rural development and the enhancement of rural residents’ subjective well-being [7].

Theoretically, the concept of social capital was widely applied by sociologists in the 1980s [8,9]. Then, social capital was viewed as the total value of individual connections (social network), and the norm of reciprocity developed from the network [10,11]. In the past few decades, social capital has been introduced to multiple disciplines, ranging from social sciences, health, and education, to sustainable development [12,13,14]. Studies have revealed the positive correlation between subjective well-being and the social capital in developed and developing countries [15,16,17,18,19,20,21,22,23]. Since the 1990s, social capital has attracted much attention from theorists on global social economic development, as a major nonmarket driver of residents’ subjective well-being and social economic development [24]. The social capital theory has been employed to evaluate the quality of life of residents, solving the defect of traditional production factors (economic capital and human capital) in explaining microscopic, individual, subjective well-being [25]. In this way, social capital becomes an important driving force for sustainable rural development, especially in developing regions [26].

In September 2018, the Chinese government issued the Strategic Plan for Rural Revitalization (2018–2022), which clearly defined the goals of rural revitalization: building rural areas with thriving businesses, pleasant living environments, social etiquette and civility, effective governance, and prosperity. The rural revitalization strategy become an important move to realize SGDs in rural China. Since its implementation, China has lifted around 100 million people out of poverty and achieved the SDG of poverty alleviation ten years in advance. This remarkable achievement is of great significance to China and the world [27]. Nevertheless, the implementation of rural revitalization strategy in China mostly emphasizes the macroscopic aspects of rural areas, such as industry, institutions, guarantees, and norms. Little attention is being paid to subjective factors of farmers [28]. However, farmers and herdsmen are the carriers and beneficiaries of sustainable rural development, as well as the evaluators of the fulfilment of rural SDGs. If the rural areas want to achieve SDGs smoothly, it is crucial to explore the subjective factors that affect the subjective well-being of farmers and herdsmen [29]. As a nonmarket force, social capital can effectively enhance residents’ subjective well-being, and play an important role in China’s pursuit of rural revitalization. However, nowadays very few scholars have examined this issue [30]. Social capital has regional features, as it varies with geographical locations and cultural environments.

The regional utility of social capital is deeply affected by production model, which directly characterizes and influences culture [31]. In northern China’s agropastoral transition zone (APTZ), rural areas witness the coexistence of two production modes, namely, farming and animal husbandry, and the intermingling of nomadic culture and farming culture. The APTZ boasts 252 million hectares of grassland, about 26.3% of the total land area in China. There are 268 counties that entirely or partly depend on animal husbandry. Seventeen ethnic minorities live in the APTZ. Their total population amounts to 14.27 million, taking up 13.4% of China’s ethnic minority population. This stronghold of ethnic minorities is also a contiguous impoverished area. It is the key difficulty in SDGs’ realization of rural China [32].

Some studies have shown that farmers and herdsmen under different production modes possess different social capitals [33]. Nonetheless, few scholars had investigated how the difference in production model influences social capital and subjective well-being, i.e., what is the role of the production model in the relationship between social capital and subjective well-being. By solving the problem, this article clarified the relationship between social capital, production model, and the subjective well-being of farmers and herdsmen. The research results promote SDGs’ realization in northern China’s APTZ, and provide a scientific reference and decision basis for similar APTZs in developing countries across the globe.

## 2. Literature Review and Theoretical Analysis

### 2.1. Literature Review

The existing studies on social capital mainly tackle its connotations and the influence of social capital on enterprises and regional economy. There has been little reported on the utility of rural social capital. Social capital is mostly understood as a guarantee of the efficiency of social organizations. Structurally, social capital can be divided into an individual layer and a collective layer. Its connotations cover three dimensions, namely, social trust, social standard, and social network [34,35]. On the individual layer, social capital usually refers to the social network connections that directly bring welfare. On the collective layer, Wouter (2014) found that social capital stands for the extended trust and cooperation in society [36]. Most empirical studies quantify social capital as social network and trust [37]. For an enterprise, Chell (2016) found that the success rate of initial public offering (IPO) can be increased if its intermediary boasts the social capital of issuance examination committee (IEC) during the examination [38]. Listed enterprises prefer to invest in regions with a high level of social capital, to form joint ventures with other enterprises, and make diversified investment. Additionally, Li (2014) took the top management team as the research case, and found that social capital and political relations are mutually replaceable in corporate investment decision making [39]. The accelerated accumulation of social capital will divert more labor flow to technological innovation, especially in high-level innovation areas [40]. Gericke (2017) took Germany as an example, and found that the diversion helps to solve government failure and market failure, and find a middle way to effective public governance [41]. Hence, the crowding-out effect of resource development on technological innovation will diminish, cutting off the pathway of resource curse. Moreover, Li (2018) found that social capital can work with technological innovation to break the resource curse [42]. Social capital is an important factor affecting farmers’ life satisfaction and well-being. Subjective well-being is more heavily influenced by the nonmonetary attributes of a rural lifestyle (safety, tranquility, community relations, etc.) [43]. Scoppa (2008) studied the determinants of subjective well-being in Italian families and found that there were higher community social capital, individual social capital, and subjective well-being. Additionally, the stock of social capital will make people happier [44]. Rodriguezpose (2014) examined social capital and residents’ subjective well-being. It was found that social capital can positively affect residents’ subjective well-being, and its main driving force is informal social interaction [45]. Bartolini (2013) believes that the decline in American residents’ subjective well-being is mainly due to the decline in residents’ communication. The impact exceeds the promotion effect caused by the increase in income [46]. The influence of social capital on well-being also confirms that the higher the social capital, the stronger the farmers’ well-being [47].

In summary, social capital has multidimensional and multilayered connotations and impacts [48]. On the multi-dimensional aspect, the previous studies have mainly measured social capital in three dimensions: social network, social trust, and social standard [49]. The first two dimensions are the focal points of the research into residents’ subjective well-being: (1) social network can significantly improve people’s welfare and enhance the subjective well-being of residents [50]. (2) Social trust reflects the sufficiency of social capital; the stronger the social trust, the higher the subjective well-being [21]. Overall, the existing research has not paid enough attention to the influence of social capital on residents’ subjective well-being in rural communities. The existing studies have tended to analyze the connotations and features of social capital from the macro and meso perspectives [51], but have rarely analyzed or summarized social capital from the micro perspective of residents. On the multi-layered aspect, scholars have not paid enough attention to the following problems: optimizing the internal structure and diversifying the improvement paths of social capital by exploring the interaction mechanisms between social capital on different layers, when rural social capital comes into play. The literature has examined the influence of social capital on residents’ subjective well-being through theoretical analysis and empirical study. However, there are several defects within the literature: (1) There is little reported on rural social capital, especially in rural areas with multiple production modes in the APTZ. (2) The influence of social capital on residents’ subjective well-being has been mostly quantified from the single dimension of social trust, or the two dimensions of social trust and social network. Social capital had not been measured in multiple dimensions, such as trust, network, and standard. (3) Few scholars have studied the interaction mechanisms between social capital on different layers in the structure of social capital. (4) Production mode has rarely been considered in the discussion of the relationship between social capital and subjective well-being.

Therefore, from the micro perspective of farmers and herdsmen, this article attempted to extend the connotations of social capital from three (social trust, social standard, and social network) to six (social network, social trust, social participation, social standard, social fame, and common vision). On this basis, a theoretical framework was established to measure social capital, which covered the six dimensions and two layers (individual and collective). Then, the mutual replaceability between individual social capital and collective social capital was discussed in terms of the influence over the subjective well-being of farmers and herdsmen. This article empirically identified the social capital difference between farmers and herdsmen under different production modes, evaluated the influence of this difference over subjective well-being, and discussed how the same amount of social capital contributes differently to the subjective well-being of farmers and herdsmen under different production models. This research fully clarified the relationship between social capital and the subjective well-being of farmers and herdsmen, and provided new theoretical and practical bases for the government to make the most of the utility of rural social capital in the pursuit of rural SDGs. Compared with the previous research, this article mainly made three distinct contributions: Firstly, the previous research on social capital was largely limited to the effects of social capital on enterprises, institutions, and economic development [27,28], failing to explore the structure and features of social capital. Focusing on the hierarchy of social capital, this article verified the mutual replaceability between individual social capital and collective social capital in the influence of subjective well-being, and provided new insights into the internal structural features of social capital and the action mechanisms of social capital on subjective well-being. Secondly, the literature generally examined the influence of two dimensions, i.e., social network and social trust, on subjective well-being [39,48,50]. In this article, a theoretical framework for social capital measurement with more dimensions of social capital were considered, including social standard, social participation, social fame, and common vision, forming a six-dimensional and two-layer framework, including an individual layer and collective layer. Thirdly, this article innovatively included production mode into the research scope. An in-depth analysis was carried out to disclose how production mode affects the influence of social capital on subjective well-being, and reveals the specific relationship between social capital and subjective well-being.

**Figure 1:**
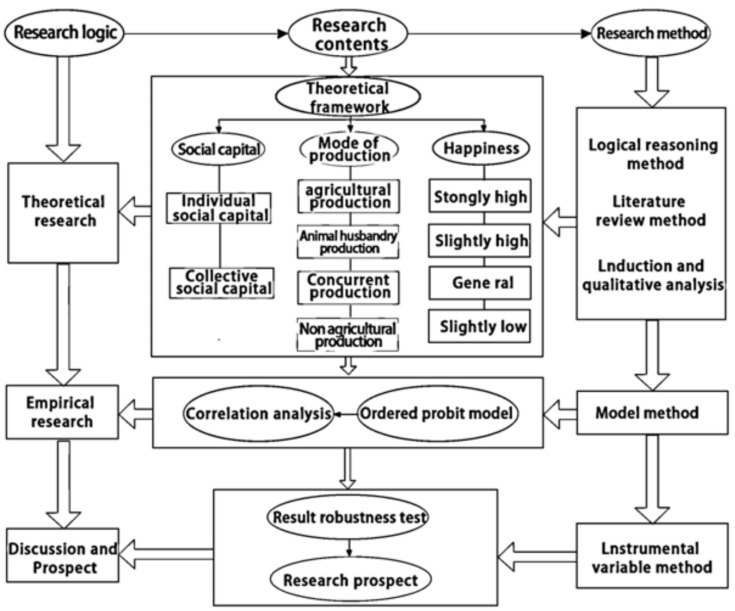
Research framework.

The remainder of this article was organized as follows: Section 2 presented the theoretical analysis framework and hypothesis based on the literature review; Section 3 introduced the methodology and data; Section 4 analyzed and tests the empirical results; Section 5 discussed the research results; Section 6 summarized research limitations and predicts the directions of future work, and put forward the conclusions and suggestions. The research framework as follows (Figure 1).

### 2.2. Theoretical Analysis

Social capital is the greatest contributor to the subjective well-being of farmers and herdsmen. It is important to identify which elements of social capital affect the subjective well-being of farmers and herdsmen [52]. There have always been two research paths toward social capital: the individual path and the collective path. Researchers taking the two paths strongly disagree on theories and concepts. To clarify the theoretical essence of social capital, it is inevitable to conduct dialogs across the individual and collective layers [52].

Our research took farmers and herdsmen as microsubjects. On the one hand, individual farmers and herdsmen enhance income, production efficiency, sense of belonging, and self-recognition (social fame), through exchanges and interactions with neighbors, relatives, village cadres, and cooperatives (social participation, and social network), thereby improving the level of subjective well-being [16]. On the other hand, the collective social environment (social trust, social standard, and common vision) indirectly affects the subjective well-being of farmers and herdsmen by influencing the income, education level, employment concept, health cognition, and risk resistance [53].

**Figure 2:**
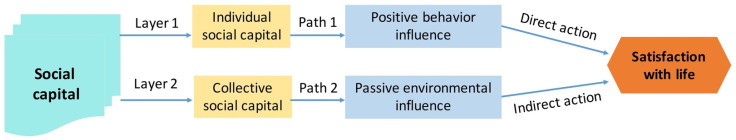
Influence paths of social capital on subjective well-being.

Therefore, this article was mainly concerned about the social capital on the layer of individual farmers and herdsmen, and the social capital on the collective layer. Theoretical and empirical studies had explored how individual social capital and collective social capital act on the subjective well-being of farmers [54]. In general, social capital can promote subjective well-being along two different paths (Figure 2): the individual path and the collective path [53]. Hence, the following hypothesis was presented:

**Hypothesis** **1** **(H1).** *Social capital promotes the subjective well-being of farmers and herdsmen.*

**Figure 3:**
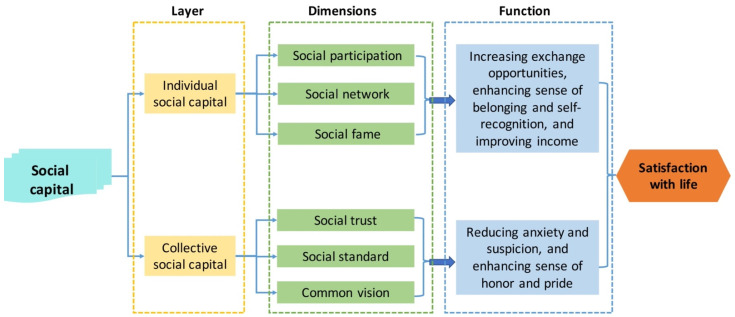
Theoretical analysis framework for the influence of social capital over the subjective well-being of farmers and herdsmen.

Individual social capital mainly improves the subjective well-being of farmers and herdsmen (Figure 3) via their social participation, social network, and social fame (Figure 3). The social participation is mainly measured by whether a subject participates in production technology trainings on agricultural and animal husbandry, collective meetings, and cadre elections of the village (or “gacha” in local dialect). Social participation benefits the farmers and herdsmen [55]. After frequent social participation, the subjects can better recognize their own production and living environments, and perceive a higher subjective well-being [55]. Social network mainly consists of the connections with relatives, village collective, and technology training departments of agricultural and animal husbandry. It can be characterized by the following indicators: the number of migrant workers, the number of neighbors in the village that often come around, the number of farmers and herdsmen in the village that are willing to lend money, monthly cellphone bill, etc. [56]. Social fame can be characterized by the number of neighbors and relatives that are willing to offer help, the influence of close relatives in the locality, the frequency of being invited to mediate conflicts between neighbors, and the respect received in the village [57].

The collective social capital indirectly acts on the subjective well-being of farmers and herdsmen via social trust, social standard, and common vision (Figure 2). Social trust mainly refers to the degree of trust in surrounding people, policies, governments, village committees, as well as agriculture and animal husbandry cooperatives. In rural society, trust is the foundation of all social activities [58]. An important way for a villager to improve subjective well-being of farmers and herdsmen is to win the trust from other villagers [59]. Social standard influences the behaviors of farmers and herdsmen through customs, ethics, and association charters, thereby affecting the subjective well-being of farmers and herdsmen [60]. Common vision primarily includes whether a subject wishes the village to get better, and whether farmers and herdsmen is willing to contribute his/her time and money to village development. The higher the common vision, the more frequent the mutual help between villagers, and the better the subjective well-being of farmers and herdsmen.

Both individual and collective social capitals impact the production and life of farmers and affect the subjective well-being of farmers and herdsmen. Our survey shows that when the individual social capital (e.g., social network) is insufficient, some farmers and herdsmen would resort to collective social capital (e.g., social trust) to improve production and life status and enhance their subjective well-being. Therefore, this article assumed that the collective social capital would play a greater role when farmers and herdsmen lack individual social capital.

**Hypothesis** **2** **(H2).** *Individual social capital and collective social capital are mutually replaceable in terms of the effect on the subjective well-being of farmers and herdsmen, that is, when the individual social capital decreases, the subjective well-being of farmers and herdsmen will depend more on collective social capital, and vice versa.*

Production mode refers to the way of acquiring the materials necessary for social life. It is the system of connections between people and nature, as well as those between people, formed through the production process [61]. There are generally two types of production modes: material production mode (method of material acquisition) and social production mode (form of social economic activities) [18]. Social capital is a specific range of social connections generated (often directly) by people through social interaction. The dimensions of social capital are not limited to formal institutions such as politics and laws and informal institutions such as social customs. The form of organizations (e.g., social relationship network) and the expression of people’s preference (e.g., trust) are also parts of social capital [62]. The production modes, both physical and social, affect the social capital features of farmers and herdsmen, exerting an indirect effect on their subjective well-being. The influence of social capital on subjective well-being varies with the production modes of farmers and herdsmen. In other words, the production mode of farmers and herdsmen determines how their subjective well-being is affected by social capital. Hence, the following hypothesis was put forward:

**Hypothesis** **3** **(H3).** *Production mode has a significant influence on the subjective well-being of farmers and herdsmen. The same amount of social capital contributes differently to the subjective well-being of farmers and herdsmen under different production models.*

The above theoretical hypotheses were empirically verified through abundant microscopic data in this article.

## 3. Methodology and Data

### 3.1. Data Sources

**Figure 4:**
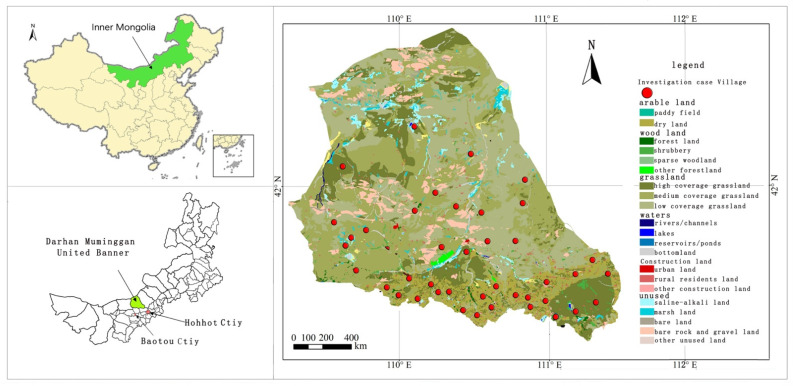
Study area and sampling points.

The study area is Darhan Muminggan Joint Banner of Inner Mongolia, located in in the northern China APTZ (Figure 4). Darhan Muminggan Joint Banner administers an area of 18,177 km^2^, including 77,007 hm^2^ of farmland and 1,597,328 hm^2^ of grassland. There are 12 towns and townships (or “sumu” in local dialect) in the study area, own 41,895 households and 111,586 people. Specifically, Bailingmiao is a town with a composite industrial structure. The residents in this town either live on both agriculture and animal husbandry (hereinafter referred to as “farmers + herdsmen” (FH)), or live on neither agriculture nor animal husbandry. Wukehudong is a town dominated by agriculture (hereinafter referred to as “non-farmers/herdsmen” (NFH)). The residents in this town live entirely on agriculture. Bayinaobao is a township dominated by animal husbandry (hereinafter referred to as “pure farmers” (PF)). The residents in this town live entirely on animal husbandry (hereinafter referred to as “pure herdsmen” (PH)). Considering the research needs, three rural surveys were carried out from July to December 2020 in Bailingmiao, Wukehudong, and Bayinaobao.

(1) Preliminary survey. In June 2020, the research team visited the government departments of Darhan Muminggan Joint Banner, including the bureaus of statistics, agriculture and animal husbandry, and land and resources, as well as the towns and townships in the banner. During the visit, the research team collected the background data on the funds, assets and resources lawfully owned by rural collective economic organizations, and carried out a questionnaire survey on a few farmers and herdsmen;
(2) Formal survey. In August 2020, a questionnaire was designed and distributed. Based on the background data and farmers/herdsmen data collected through the preliminary survey, the questionnaire was improved and used for the sampling survey;
(3) Additional survey. In October 2020, an additional survey and a field test were conducted to solve the incomplete data of the responses and verify the analysis results. A total of 732 (94%) valid responses was obtained. The respondents and sampling points are given in the appendix.

### 3.2. Division of Production Modes

Referring to the previous divisions of the production mode for farmers and herdsmen [25,33], and the production and life of rural farmers and herdsmen in the APTZ, four production modes were defined: PF, PH, FH (proportion of agricultural and animal husbandry income ≥85%, and the proportion of agricultural income or animal husbandry income ≥35%), and NFH (the proportion of nonagricultural and animal husbandry income ≥85%) (Table 1).

### 3.3. Measurement of Social Capital

(1) Measuring system of social capital

Theoretical analysis shows that both individual and collective social capitals affect the subjective well-being of farmers and herdsmen. Hence, this article divided the social capital of farmers and herdsmen into individual and collective social capitals. Specifically, individual social capital covered 16 indicators in the three dimensions of social participation, social network, and social fame, while collective social capital covered 13 indicators in the three dimensions of common vision, social trust, and social standard. In total, social capital was measured by 29 indicators (Table 2). Each indicator was valued against a five-point Likert scale. The six-dimensional items of the questionnaire were subjected to confirmatory factor analysis (CFA) to verify the feasibility of our model. The convergent and discriminant validity between variables, and composite reliability between items, were all greater than 0.8, the average variance extracted (AVE) was greater than 0.6, and the square root of the AVE was above the correlation coefficient between a variable and any other variable. The results demonstrate a high convergent and discriminant validity between the dimensions (Table 2).

(2) Social capital index

For each dimension of social capital, the scores of relevant indicators were added up and averaged to obtain the index of that dimension. The social capital index of farmers and herdsmen is the weighted average of the indicators of all six dimensions, with contribution rate (Table 2) as the weight:

(1) $$B_{d}=\frac{1}{n}\sum_{i=1}^{n}A_{j}$$

(2) $$B=\sum_{d=1}^{n}B_{d}*C_{d}$$

where *B* is the social capital index of farmers and herdsmen; *B_d_* is the value of dimension *d* in the social capital measuring system; *A_j_* is the normalized score of item *j* in dimension *d*; *n* is the number of items in dimension *d*; *C_d_* is the weight of dimension *d*. The individual social capital index and collective social capital index were calculated in a similar manner.

### 3.4. Model Setting and Testing

(1) Model setting

In the theoretical hypotheses, the explained variable was a binary variable; the key explanatory variable, subjective well-being, was measured by an ordered Likert scale; the other key explanatory variable, social capital, was also very orderly. Therefore, the multivariate ordered probit model was selected to test the three hypotheses. Three basic test models were established:

Model 1:

LifeS = α + β_1_SocialC + β_2_Participation + β_3_Network + β_4_Fame + β_5_Trust + β_6_Vison + β_7_Standard + β_8_HumanC_controls_ + β_9_NatureC_controls_ + β_10_MaterialC_controls_ + β_11_FinancialC_controls_ + ε (3)

Model 2:

LifeS = α + β_1_individualSC × collectiveSC + β_2_IndividualSC × (1−collectiveSC) + β_2_HumanC_controls_ + β_3_NatureC_controls_ + β_4_MaterialC_controls_ + β_5_FinancialC_controls_ + ε (4)

Model 3:

LifeS = α + β_1i_P_i_ + β_2i_SocialC_pi_ + ε (5)

where, LifeS is subjective well-being; SocialC is social capital; Participation, Network, Fame, Trust, Vision, and Standard are social participation, social network, social fame, social trust, common vision, and social standard, respectively; HumanC, NatureC, MaterialC, and FinancialC are human capital, nature capital, material capital, and financial capital, respectively.

The name and meaning of each variable in the three models are given in Table 1 and Table 2. Drawing on the relevant literature and the sustainable livelihood framework of British Department for International Development (DFID), human capital, material capital, financial capital, and nature capital were selected as control variables (Table 3).

(2) Explained variable

The explained variable was the subjective well-being of farmers and herdsmen. It was measured by an orderly scale (1 = strongly unsatisfied, 2 = unsatisfied, 3 = neutral, 4 = satisfied, 5 = strongly satisfied) in our questionnaire. The survey showed the mean and standard deviation of the explained variable were 2.76 and 0.435, respectively. Therefore, the subjective well-being of farmers and herdsmen was not highly dispersed, but oscillates about the mean. The farmers and herdsmen in the study area did not have a high subjective well-being.

(3) Explanatory variables

Social capital. As mentioned before, social capital was divided into individual social capital and collective social capital. The former consisted of 16 indicators in the three dimensions of social participation, social network, and social fame, and the latter consisted of 13 indicators in the three dimensions of social trust, common vision, and social standard (Table 2).

Production mode. The farmers and herdsmen were divided into four production modes: PF, PH, FH, and NFH.

(4) Control variables

This article classified the livelihood of farmers and herdsmen according to the DFID’s sustainable livelihood framework. Inspired by the results of Wang Changhai (2017) and Li Wenlong (2019), the influence of social capital on the subjective well-being of farmers and herdsmen was measured with human capital, nature capital, material capital, and financial capital as control variables.

(5) Model testing

Model 1 tested the influence of social capital on the subjective well-being of farmers and herdsmen. The goal was to verify whether social capital has a significant and positive impact on the latter. Our hypothesis held that social capital significantly promotes the subjective well-being of farmers and herdsmen. If the hypothesis holds, *β*_1_ must be significantly positive in model 1.

Model 2 tested whether individual social capital and collective social capital were mutually replaceable in their influence over the subjective well-being of farmers and herdsmen, that was, whether the subjective well-being of farmers and herdsmen depended more on collective social capital when the individual social capital decreased, and whether it depended more on individual social capital when the collective social capital decreased. Our hypothesis held that individual social capital and collective social capital were mutually replaceable in terms of the effect on the subjective well-being of farmers and herdsmen. If the hypothesis holds, the coefficient *β*_1_ of the cross-term Individual SC × Collective SC must be much smaller than the coefficient *β*_1_ of the cross-term Individual SC × (1−Collective SC) in model 2, i.e., the farmers and herdsmen with a low individual social capital depended more on collective social capital.

Model 3 tested whether production model had a significant impact on the subjective well-being of farmers and herdsmen, and whether the same amount of social capital contributed differently to the subjective well-being of farmers and herdsmen under different production models, that was, when the farmers and herdsmen had the same social capital, would social capital contribute differently to the subjective well-being of farmers and herdsmen under four different production modes (Social C_P_i_). Our hypothesis held that production mode had a significant influence on the subjective well-being of farmers and herdsmen. The same amount of social capital contributed differently to the subjective well-being of farmers and herdsmen under different production models. If the hypothesis holds, the coefficient *β*_1i_ for the influence of production mode on subjective well-being must be significant, and the coefficients *β*_2i_ for the influence of different production modes must differ significantly.

## 4. Results and Analysis

### 4.1. Descriptive Statistical Analysis

(1) Subjective well-being under different production modes

**Figure 5:**
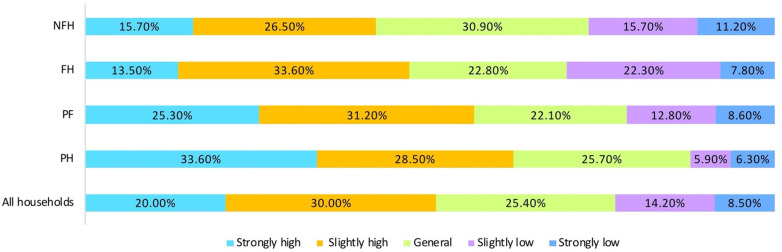
Status quo of subjective well-being of farmers and herdsmen.

The survey results showed that the farmers and herdsmen generally had a low subjective well-being, and the subjective well-being of farmers and herdsmen varies greatly with production modes. In general, the farmers and herdsmen with a single livelihood means have a higher subjective well-being than those with diverse livelihood means (Figure 5). The results of descriptive statistics agreed with our hypothesis that the farmers and herdsmen under different production modes differ in subjective well-being.

(2) Social capital features of farmers and herdsmen under four production modes

A chi-squared test for multiple samples was carried out on the social capital of farmers and herdsmen under four production modes. The chi-squared values of the different dimensional indicators for social capital were 20.113, 31.421, 16.248, 61.352, 70.175, 46.273, and 101.25, and the corresponding *p*-value was 0.000, below the significance level of 0.001. The results confirmed that farmers and herdsmen under different production modes differ significantly in social capital (Table 4).

(3) Descriptive statistical analysis on main variables

For the lack of space, only the three key findings from Table 2 are reported here: Firstly, social capital had a significant positive correlation with subjective well-being, which was in line with our expectation. Secondly, there was a significant negative correlation between individual social capital and collective social capital, i.e., the two factors were mutually replaceable, as suggested by our theoretical hypothesis. Thirdly, social capital had a significant positive correlation with subjective well-being of farmers and herdsmen under different production modes, and the correlation coefficients vary with the production modes. This also meets our hypothesis. All findings were empirically analyzed in the subsequent sections.

### 4.2. Analysis of Regression Results

The empirical analysis of the three models was carried out with a multivariate ordered probit model using Stata 14.0, aiming to test the influence of social capital on the subjective well-being of farmers and herdsmen. The multivariate ordered probit model is an ideal tool for handling orderly explained variables. It has been widely applied in the regression analysis of multiclass variables, especially subjective well-being. During model estimation, this article improved the accuracy of statistics by solving the autocorrelation, heteroscedasticity, and multicollinearity of the data.

(1) Influence of social capital on the subjective well-being of farmers and herdsmen

Table 5 listed the statistics on whether social capital has a significant influence on the subjective well-being of farmers and herdsmen, and the coefficients (*β*) and directions of that influence.

After controlling human, nature, material, and financial capitals, it was learned that social capital has a significant positive correlation with subjective well-being of farmers and herdsmen, that was, the farmers and herdsmen owning more social capital have a higher subjective well-being. Each unit of increase in social capital improved their subjective well-being by 65.3%.

Social network was the leading contributor among the dimensions of individual social capital, while social trust was the leading contributor among the dimensions of collective social capital. Overall, social network had a greater impact on the subjective well-being of PF than any other dimension of social capital. For PH, FH, and NFH, the greatest impactor was social trust. By contribution to the subjective well-being of farmers and herdsmen, the six dimensions of social capital can be ranked as social network (1.812) > social trust (1.756) > social participation (1.654) > social standard (1.605) > social fame (0.597) > common vision (0.412).

The six dimensions acted differently on subjective well-being of farmers and herdsmen under different production modes. Specifically, social participation contributed the most to the subjective well-being of PF (0.778); social network contributed the most to the subjective well-being of FH (1.955); social fame contributed the most to the subjective well-being of PH (0.508); social trust contributed the most to the subjective well-being of NFH (1.717); common vision contributed the most to the subjective well-being of PH (0.631); social standard contributed the most to the subjective well-being of FH (0.778).

By influence on the subjective well-being of farmers and herdsmen, the four control variables can be ranked in descending order as financial capital (2.388) > material capital (2.125) > nature capital (1.833) > human capital (1.693). From the influence of these control variables, it can be derived that the subjective well-being of farmers and herdsmen increases with the health level of laborers, education level of adult laborers, number of adult laborers in the household, per-capital annual income of the household, house, livestock, production and living equipment, and per-capita area of grassland (Table 6).

(2) Mutual replaceability between individual and collective social capitals in the influence of subjective well-being of farmers and herdsmen.

Table 6 reported the regression results of the mutual replaceability between individual and collective social capitals in the influence of subjective well-being of farmers and herdsmen. The statistics on the mutual replaceability, as well as the significance coefficients (*β*) and their directions, have been listed.

After controlling for human, nature, material, and financial capitals, it was learned that individual and collective social capitals were mutually replaceable in the influence of subjective well-being of farmers and herdsmen, that was, the coefficient *β*_1_ (0.704) of the cross-term Individual SC × Collective SC is significantly smaller than coefficient *β*_2_ (1.892) of the cross-term Individual SC × (1−Collective SC). Hence, the farmers and herdsmen with a low individual social capital depended more on collective social capital.

(3) Influence of the same amount of social capital on the subjective well-being of farmers and herdsmen under different production modes.

Table 6 listed the influence, significance coefficients (*β*) and their directions of the same amount of social capital on the subjective well-being of farmers and herdsmen under different production modes.

In the APTZ, the farmers and herdsmen had a relatively low subjective well-being. The subjective well-being of 32% of all households was slightly high or strongly high. However, production mode significantly promoted the subjective well-being of farmers and herdsmen at the level of 1%.

Meanwhile, the same amount of social capital contributed differently to the subjective well-being of farmers and herdsmen under different production models. The greatest contribution rate (33.9%) to subjective well-being was observed under PH, followed in turn by 24.9% under PF, 15.9% under NFH, and 13.4% under FH. Overall, the subjective well-being decreased gradually from PH, PF, NFH, to FH. Therefore, the farmers and herdsmen with a single livelihood means had higher subjective well-being than those with diverse livelihood means.

### 4.3. Robustness Test

This article studied the influence of social capital on the subjective well-being of farmers and herdsmen. There might be a missing variable that simultaneously impacted social capital and subjective well-being, causing the two to exhibit a positive correlation. Additionally, reverse causality might disrupt the test on the mutual replaceability between individual and collective social capitals; the growth of individual social capital could be driven by the increase in collective social capital.

The instrumental variables (IV) method was selected to solve the above problems. The age of farmers and herdsmen was taken as an instrumental variable. Age was significantly correlated with individual health, which in turn has a significant relationship with social capital [63]. Thus, age must be related to social capital: people in different age groups own different amounts of social capital, and seniors tend to possess relatively lower social capital [64]. Most importantly, age has nothing to do with the subjective well-being of residents [62]. That is why age was chosen as the instrumental variable to solve the problems of missing variable and reverse causality.

Table 7 displays the empirical test results of models 1 and 2 using the IV method. According to the last three rows, the effective test variables, i.e., partial R^2^ and partial F (*p*-value), of the instrumental variable both fell within the effective range. The results indicated that age was an effective instrumental variable, and the test results agreed with our conclusions. In other words, our research has drawn robust conclusions.

## 5. Discussion

The research results suggested that social capital has a stable and significant positive correlation with residents’ subjective well-being. This was consistent with the result of previous research [52]. However, the social capital contributed differently to residents’ subjective well-being under different production modes. This finding revealed the underlying action mechanism of social capital on residents’ subjective well-being.

In general, the social network dimension of individual social capital and the social trust dimension of collective social capital made outstanding contributions to the subjective well-being of farmers and herdsmen. The possible reasons were as follows: it was difficult to expand the social network in rural areas of the APTZ, due to the low density and scattered distribution of the rural population; the trust and cooperation between farmers and herdsmen were undermined by market policies such as the double contract system of grassland and livestock.

The subjective well-being under PF was primarily affected by the social network, and that under PF, FH, and NFH was mainly driven by social trust. This was attributable to the relatively backward agricultural infrastructure in the APTZ. A stronger social network was needed to generate the agglomeration effect, which benefited the sales and saved the transport cost of agricultural products. Influenced by traditional nomadic culture, PH, FH, and NFH subjects had a strong sense of belonging. Thus, their subjective well-being was significantly affected by social trust. To better enhance their subjective well-being, the farmers and herdsmen should be supported in different dimensions of social capital, depending on their production modes.

It was also found that individual and collective social capitals were mutually replaceable in enhancing the subjective well-being of farmers and herdsmen. This was probably the result of the rational choice of farmers and herdsmen to produce and live smoothly. The farmers and herdsmen often sought help from the collective when their development was constrained by capital shortage. However, when individual social capital was sufficient for production and living, the residents often did not rely on collective social capital. Therefore, the implementation of rural revitalization strategy needs to maximize the positive effect of collective social capital on the subjective well-being of farmers and herdsmen. This poses a sharp contrast with the traditional research, which only stresses the influence of individual social capital on the subjective well-being of farmers and herdsmen [33,65].

Our research also revealed that high collective social capital increased the degree of social organization, making up the negative effect of individual social capital shortage on the subjective well-being of farmers and herdsmen. This revelation opened new paths to supporting subjective well-being. The subjective well-being of farmers and herdsmen could be enhanced by providing good rural community norms, a strong trust, and a high sense of belonging.

Furthermore, it was observed that the same amount of social capital contributed differently to the subjective well-being of farmers and herdsmen under different production modes. The greatest contribution occurred under PH, followed in turn by FH, NFH, and FH. This was probably the combined outcome of spatial factors and production model. PH and PF subjects lived far from cities and were scattered across the rural areas. They could only acquire a limited amount of social capital. On the contrary, NFH and FH subjects enjoyed a location advantage: they lived in the peripherals of cities, and close to each other. As a result, these subjects maintained a developed social network and had more chances to access social capital. Therefore, the crux of enhancing the subjective well-being of farmers and herdsmen in APTZ improved the social capital of PH and PF subjects.

The research results demonstrated that social capital, as a norm of shared resources produced by social structure and social network, offered a potential measure to mitigate the negative effect of production mode difference on subjective well-being. On the one hand, collective and individual social capitals were mutually replaceable in the influence of subjective well-being of farmers and herdsmen. Thus, collective social capital can be increased to make up for the negative impact of individual social capital shortage on the subjective well-being of farmers and herdsmen. On the other hand, the production mode difference significantly affected the action of social capital on the subjective well-being of farmers and herdsmen. Hence, the subjective well-being of farmers and herdsmen can be elevated effectively if pertinent policies are proposed, by pinpointing the social capital demand features of different types of farmers and herdsmen. These policies could also promote sustainable rural development in the APTZ.

The stock of farmers’ and herdsmen’s social capital had a significant positive impact on their subjective well-being, and indeed propelled sustainable rural development. During the pursuit of rural SDGs, China should not only consider the construction of macroscopic hardware environments (including industries and infrastructure), but should also roll out policies and measures to improve the subjective well-being from the micro perspective of farmers and herdsmen. To enhance the subjective well-being of farmers and herdsmen, the government should prepare policies to encourage rural culture construction and boost villagers’ social capital. Two issues must be considered to improve the social capital of farmers and herdsmen: firstly, the internal hierarchy of farmers’ and herdsmen’s social capital must be optimized according to the mutual replaceability between different layers of social capital; secondly, the utility of social capital should be maximized by creating pertinent policies according to the relationship between subjective well-being and the heterogeneity of the social capital of farmers and herdsmen under different production modes.

## 6. Conclusions

This article focused on the interaction between different layers of social capital and residents’ subjective well-being under different production modes, and enriched the understanding of the interaction mechanisms based on survey data on rural farmers and herdsmen in a typical APTZ. This research discovered that social capital influenced the subjective well-being of farmers and herdsmen very differently under different production modes. More importantly, it was learned that collective social capital eliminates the negative effect of individual social capital shortage on the subjective well-being of farmers and herdsmen. This finding was particularly meaningful for rural areas in the APTZ, which were often strongholds of ethnic minorities, and contiguous impoverished areas with a fragile ecological environment and a frequent occurrence of draughts. For these areas, the findings opened new paths to improve the subjective well-being of the locals, and provided a reference for developing rural areas with fragile ecological environments around the world.

There are several limitations of this research. Firstly, the social capital of individual households and rural communities was obtained through a questionnaire survey. Although this method is widely adopted by researchers, the survey results are affected by individual features of the respondents [66]. The reliability of the rating criteria is easily influenced by the number of interviewees. Secondly, this article expounds on the influence of production model over the interaction between social capital and residents’ subjective well-being. However, the specific factors affecting the production mode were not discussed in detail. Thirdly, the authors examined the influence of individual and collective social capitals on subjective well-being, yet failed to clarify the structural impacts of social capital layers on residents’ subjective well-being. In addition, although this study discusses the influence of individual social capital and collective social capital on well-being, the structural impact of social capital on residents’ well-being is not clear. Therefore, based on the research conclusions, we will further study the interaction relationships, interaction path and mechanisms among residents’ production mode, social capital and well-being, which will help clarify the interaction logic of residents’ production mode, social capital and well-being, and put forward targeted measures to improve residents’ well-being. Furthermore, studying the impact of the hierarchical structure of individual social capital and collective social capital on residents’ well-being, revealing the impact law of social capital on residents’ well-being, and exploring the internal mechanisms of social capital affecting residents’ well-being, will help to deepen the connotation of these impacts.

## Figures and Tables

**Table 1:** Descriptive indicators of the four production modes.

	PF		PH		FH		NFH		Median	Chi-Squared
	Mean	Standard Deviation	Mean	Standard Deviation	Mean	Standard Deviation	Mean	Standard Deviation		
Age of household owner	2.320	0.570	2.260	0.760	2.370	0.620	2.200	0.600	2.10	31.22 *
Education level of household owner	0.501	0.210	0.420	0.210	0.540	0.240	0.580	0.250	0.40	20.40 *
Household size (people/household)	3.602	1.340	3.310	1.530	3.80	1.230	3.900	1.770	3.50	21.73 **
Labor proportion (%)	68.726	21.910	65.620	20.430	59.850	21.340	56.460	16.840	60.03	8.242 **
Per-capita annual income (10,000 yuan/person)	1.682	0.861	1.723	0.794	1.832	0.821	1.712	0.543	1.426	77.45 **
Engel coefficient	0.332	0.151	0.323	0.162	0.306	0.153	0.262	0.141	0.310	59.38 **
Sample size	244		134		196		158			

Note: for the age of household owner, 1 point is assigned if the age is below 35, 2 if the age is between 35 and 55, 3 if the age is between 55 and 70, and 4 if the age is above 4; For the education level of household owner, 0 point is assigned if the owner is illiterate, 0.25 if the owner is a graduate of primary school, 0.5 if the owner is a graduate of junior high school, 0.75 if the owner is a graduate of senior high school/secondary technical school, and 1 if the owner is a graduate of junior college/ordinary college and above; ** and * represent the significance at the levels of 5% and 10%, respectively.

**Table 2:** Descriptive statistics of social capital indicators.

Layers	Dimensions	Indicators	Values	Mean	Standard Deviation	Cronbach’s Alpha
Individual social capital	Social participation	Frequency of participating in production and life activities organized by the government (P1)	1 (no participation)~5 (frequent participation)	1.26	1.531	0.772
		Frequency of participating in village cadre election (P2)		2.37	1.620	
		Degree of recognition for village cadre election (P3)	1 (no recognition)~5 (high recognition)	2.37	1.384	
	Social network	Communication frequency with intimate friends (I1)	1 (very few)~5 (many)	3.29	0.973	0.768
		Communication frequency with relatives living in other places (I2)		2.11	0.654	
		Degree of connection with village cadres (I3)		1.88	0.901	
		Learning frequency at technology training departments of agricultural and animal husbandry (I4)		1.60	0.857	
		Number of migrant workers (I5)		2.55	1.026	
		Number of households in the village that often come around (I6)		3.58	1.021	
		Number of villagers willing to lend money (I7)		4.01	1.641	
		Monthly cellphone bill (I8)		3.42	1.09	
	Social fame	Number of people nearby willing to offer help during the busy season (F1)	1 (very few)~5 (many)	2.01	0.823	0.789
		Frequency of participating in weddings and funerals of other villagers (F2)		3.02	0.991	
		Influence of close relatives in the locality (F3)		2.37	1.546	
		Frequency of being invited to mediate others’ conflicts (F4)		1.68	1.547	
		Respect received in the village (F5)		1.82	1.783	
Collective social capital	Social trust	Trust of relatives and friends (T1)	1 (totally disagree)~5 (totally agree)	3.02	0.796	0.756
		Belief in national policies on TV and the internet (T2)		4.05	1.265	
		No connection needed to handle an affair at any government agency (T3)		2.88	1.248	
		Trust in village committee (T4)	1 (extreme distrust)~5 (strong trust)	3.26	1.035	
		Trust in cooperative (T5)		3.22	1.043	
	Common vision	Most people willing to help each other (V1)	1 (totally disagree)~5 (totally agree)	3.11	1.254	0.735
		Care about events in the village and neighboring villages (V2)		4.35	0.963	
		Wish the village to get better (V3)		3.55	1.569	
		Willing to contribute time and money to village development (V4)		3.56	1.084	
	Social standard	Full compliance with village regulations and nongovernmental agreements (S1)	1 (totally disagree)~5 (totally agree)	2.67	1.045	0.775
		Low frequency of theft in the village (S2)		3.01	0.937	
		Rare occurrence of quarrels in the village (S3)		2.54	0.986	
		Safe living in the village (S4)		3.81	1.182	

**Table 3:** Variable definitions and calculation methods.

Primary Indicators	Secondary Indicators	Rating Criteria	Mean	Standard Deviation	Sign (−/+)
Explained variable					
Subjective well-being		1 = strongly unsatisfied, 2 = unsatisfied, 3 = neutral, 4 = satisfied, 5 = strongly satisfied	2.76	0.435	
Explanatory variables					
Social capital	Social participation (p), social network (I), social fame (F), social trust (T), common vision (V), social standard (S)	Social capital of farmers and herdsmen refined into 29 indicators (Table 2) in six dimensions: social participation, social network, social fame, social trust, common vision, and social standard	3.25	0.763	+
Production mode	PF(P1), PH(P2), FH (P3), NFH(P4)	1 = all income from agriculture; 2 = all income from animal husbandry; 3 = agriculture and animal husbandry income/total income ≥90%; 4 = non-agriculture and animal husbandry income/total income ≥90%	2.37	1.126	+
Control variables					
Human capital	Number of adult laborers in the household (H1)	5 (1); 4 (0.8); 3 (0.6); 2 (0.4); 1 (0.2)	0.56	0.678	+
	Education level of adult laborers (H2)	Illiterate (0); primary school graduate (0.25); junior high school graduate (0.5); senior high school/secondary technical school graduate (0.75); junior college/ordinary college and above graduate (1)	0.48	0.181	+
	Health level of laborers (H3)	Strongly healthy (1); slightly healthy (0.8); general (0.5); slightly unhealthy (0.2); strongly unhealthy (0)	0.65	0.971	+
Nature capital	Per-capita area of farmland (mu) (N1)	N1 > 100 mu (1); 70 mu < N1 ≤ 100 mu (0.8); 40 mu < N1 ≤ 70 mu (0.6); 20 mu < N1 ≤ 40 mu (0.4); 10 mu < N1 ≤ 20 mu (0.2); N1 < 10 mu (0.1)	0.68	0526	+
	Per-capita area of grassland (mu) (N2)	N2 > 5000 mu (1); 3000 mu < N2 ≤ 5000 mu (0.8); 1000 mu< N2 ≤ 3000 mu (0.6); 500 mu< N2 ≤ 1000 mu (0.4); 100 mu< N2 ≤ 500 mu (0.2); N2 < 100 mu (0)	0.79	0.892	+
Material capital	House M1 (RMB yuan)	Present value of the house based on quality and age (P1): P1 > 200,000 (1); 150,000 < P1 ≤ 200,000 (0.8); 100,000 < P1 ≤ 150,000 (0.6); 50,000 < P1 ≤ 100,000 (0.4); 10,000 < P1 ≤ 50,000 (0.2); P1 < 10,000 (0)	0.27	0.165	+
	Livestock M2 (RMB yuan)	Present value based on the number of horses, cattle, and pigs (P2): P2 > 10,000 (1); 5000 < P2 ≤ 10,000 (0.8); 3000 < P2 ≤ 5000 (0.6); 1000 < P2 ≤ 3000 (0.4); 5000 < P2 ≤ 1000 (0.2); P2 < 5000 (0)	0.504	0.604	+
	Production and living equipment M3 (RMB yuan)	Present value of the cars, tractors, motorcycles, seeders, and electric appliances owned by farmers and herdsmen (P3): P3 > 40,000 (1); 30,000 < P3 ≤ 40,000 (0.8); 20,000 < P3 ≤ 30,000 (0.6); 10,000 < P3 ≤ 20,000 (0.4); 5000 < P3 ≤ 10,000 (0.2); 1000 < P3 ≤ 5000 (0.1); P3 < 1000 (0)	0.90	1.214	+
Financial capital	Per-capital annual income of the household F1 (10,000 yuan)	Per-capita income of farmers and herdsmen in 2017	1.30	0.734	+
	Loan (yes/no) F2	0 = no, 1 = yes	0.73	0.432	+

**Table 4:** Social capital features of farmers and herdsmen.

Dimensions	PF			PH			FH			NFH			Chi-Squared
	Mean	Standard Deviation	Gini Coefficient	Mean	Standard Deviation	Gini Coefficient	Mean	Standard Deviation	Gini Coefficient	Mean	Standard Deviation	Gini Coefficient	
Social participation (P)	3.39	0.696	0.01	2.95	1.4	0.1	3.53	1.391	0.06	2.43	0.753	0.10	20.113
Social network (I)	3.76	0.84	0.06	3.19	0.628	0.06	3.94	0.738	0.08	3.22	0.872	0.12	31.421
Social fame (F)	3.75	1.198	0.04	2.26	0.892	0.11	3.83	0.968	0.11	2.23	0.71	0.08	16.248
Social trust (T)	3.47	0.906	0.05	3.05	0.655	0.08	3.72	0.985	0.07	2.45	1.155	0.07	61.352
Common vision (V)	2.94	0.999	0.05	3.44	1.02	0.05	3.05	0.948	0.10	2.32	1.231	0.10	70.175
Social standard (S)	3.16	0.475	0.06	2.65	0.390	0.14	2.87	0.715	0.08	2.98	1.052	0.10	46.275
Social capital index	3.45	0.67	0.01	3.15	0.647	0.10	3.6	0.782	0.06	2.61	0.684	0.10	101.26
Individual social capital	3.13	0.54	0.02	3.10	0.623	0.09	3.45	0.765	0.05	2.45	0.643	0.08	100.13
Collective social capital	3.77	0.56	0.01	3.20	0.657	0.11	3.75	0.732	0.07	2.77	0.676	0.12	102.32

**Table 5:** Results of hypothesis tests.

Explained Variables		Influence of Each Dimension of Social Capital on Subjective Well-Being	Mutual Replaceability between Individual Social Capital and Collective Social Capital	Influence of Social Capital on Subjective Well-Being under Different Production Modes
Estimation method		multivariate ordered probit model		
Social capital		0.653 *** (5.78)		
Participation		1.605 ** (3.87)		
Network		1.812 *** (5.86)		
Fame		0.597 ** (2.45)		
Trust		1.756 *** (5.87)		
Vison		0.412 ** (2.53)		
Standard		1.654 ** (2.47)		
Individual social capital × Collective social capital			0.704 (0.906)	
Individual social capital × (1−Collective social capital)			1.892 *** (5.86)	
Social C_P1_				0.339 *** (5.61)
Social C_P2_				0.249 *** (5.46)
Social C_P3_				0.159 *** (5.23)
Social C_P4_				0.134 *** (5.12)
Human capital	Number of adult laborers in the household (H1)	0.381 ** (2.16)	0.291 * (2.16)	0.291 * (2.16)
	Education level of adult laborers (H2)	0.701 *** (3.86)	0.604 ** (2.87)	0.604 ** (2.87)
	Health level of laborers (H3)	0.692 ** (2.76)	0.597 *** (3.16)	0.597 *** (3.16)
Nature capital	Per-capita area of farmland (mu) (N1)	1.361(1.69)	1.004 * (2.39)	1.004 * (2.39)
	Per-capita area of grassland (mu) (N2)	0.472 ** (2.23)	0.428 ** (2.73)	0.463 ** (2.21)
Material capital	House M1 (RMB yuan)	0.762 ** (2.56)	0.071 * (2.06)	0.972 * (2.16)
	Livestock M2 (RMB yuan)	0.562 * (2.01)	0.259 * (2.61)	0.452 * (2.08)
	Production and living equipment M3 (RMB yuan)	0.801 * (2.12)	0.403 ** (2.92)	0.509 ** (2.87)
Financial capital	Per-capital annual income of the household F1(10,000 yuan)	0.903 *** (4.96)	0.873 ** (2.96)	0.956 ** (3.12)
	Loan (yes/no) F2	1.485 (0.78)	0.155 (0.94)	0.843 (1.05)
Year		2018	2018	2018
Observations		1213	1213	1213
Pseudo R^2^		0.601	0.630	0.573

Note: ***, **, and * represent the significance at the levels of 1%, 5%, and 10%, respectively; the bracketed number under each coefficient is the t-statistic; Social C_P1_- Social C_P4_ are the social capital possessed by farmers and herdsmen under PH, PF, NFH, and FH, respectively.

**Table 6:** Subjective well-being of farmers and herdsmen under four production modes.

Production Mode	PF	PH	FH	NFH
	Statistic	Statistic	Statistic	Statistic
Participation	0.778 ** (3.86)	0.405 *** (5.47)	0.612 *** (4.67)	0.628 *** (5.96)
Network	1.812 *** (5.86)	1.651 *** (5.99)	1.955 ***(4.47)	1.817 *** (5.47)
Fame	0.415 ** (2.56)	0.508 ** (2.56)	0.234 ** (2.67)	0.311 ** (2.54)
Trust	1.212 *** (5.94)	1.707 *** (5.56)	1.634 *** (5.49)	1.717 *** (4.77)
Vison	0.509 ** (2.67)	0.631 * (2.17)	0.447 * (2.18)	0.514 * (2.08)
Standard	0.612 ** (2.43)	0.688 *** (5.54)	0.778 *** (4.59)	0.668 *** (5.98)
Year	2018	2018	2018	2018
Observations	491	193	278	251
Pseudo R^2^	0.641	0.545	0.621	0.585

Note: ***, **, and * represent the significance at the levels of 1%, 5%, and 10%, respectively; the bracketed number under each coefficient is the t-statistic.

**Table 7:** Robustness test results by IV method.

Explained Variables		Influence of Each Dimension of Social Capital on Subjective Well-Being	Mutual Replaceability between Individual Social Capital and Collective Social Capital	Influence of Social Capital on Subjective Well-Being under Different Production Modes
Social C		0.0032 *** (2.78)		
Individual social capital × Collective social capital			0.132 (1.306)	
Individual social capital × (1-Collective social capital)			0.0015 *** (3.65)	
Social CP1				0.0053 *** (3.78)
Social capital P2				0.0064 *** (3.46)
Social capital P3				0.0078 *** (3.23)
Social capital P4				0.0072 *** (5.12)
Human capital	Number of adult laborers in the household (H1)	0.0361 ** (2.56)	0.0667 * (2.02)	0.0671 * (2.01)
	Education level of adult laborers (H2)	0.0031 *** (3.43)	0.0481 ** (2.84)	0.0312 ** (2.96)
	Health level of laborers (H3)	0.0345 ** (2.81)	0.0064 *** (3.34)	0.0058 *** (3.46)
Nature capital	Per-capita area of farmland (mu) (N1)	0.2764 (0.97)	0.0812 * (2.17)	0.0709 * (2.21)
	Per-capita area of grassland (mu) (N2)	0.0476 ** (2.53)	0.0489 ** (2.83)	0.0432 ** (2.45)
Material capital	House M1 (RMB yuan)	0.0489 * (2.87)	0.0762 * (2.21)	0.0654 * (2.34)
	Livestock M2 (RMB yuan)	0.0804 * (2.12)	0.0589 * (2.54)	0.0724 * (2.01)
	Production and living equipment M3 (RMB yuan)	0.0895 * (2.10)	0.0442 ** (2.32)	0.0401 ** (2.56)
Financial capital	Per-capital annual income of the household F1 (10,000 yuan)	0.0021 *** (3.87)	0.0376 ** (2.46)	0.0403 ** (2.72)
	Loan (yes/no) F2	0.8012 (1.78)	0.5034 (1.03)	0.5734 (1.26)
Year		2018	2018	2018
Observations		1213	1213	1213
Pseudo R^2^		0.421	0.456	0.367
Partial R^2^		0.6572	Partial F(*p*-value)	1754 (0.000)

Note: ***, **, and * represent the significance at the levels of 1%, 5%, and 10%, respectively; the bracketed number under each coefficient is the t-statistic.

## Data Availability

The data presented in this study are available on request from the corresponding author.

## References

[1] Xu Z., Chau S.N., Chen X., Zhang J., Li Y., Dietz T., Wang J., Winkler J.A., Fan F., Huang B. (2020). Assessing progress towards sustainable development over space and time. Nature.

[2] Bryan B.A., Gao L., Ye Y., Sun X., Connor J.D., Crossman N.D., Stafford-Smith M., Wu J., He C., Yu D. (2018). China’s response to a national land-system sustainability emergency. Nature.

[3] United Nations (2015). Transforming Our World: The 2030 Agenda for Sustainable Development. https://www.unfpa.org/resources/transforming-our-world-2030-agenda-sustainable-development.

[4] Liu J., Mooney H., Hull V., Davis S.J., Gaskell J., Hertel T., Lubchenco J., Seto K.C., Gleick P., Kremen C. (2015). Systems integration for global sustainability. Science.

[5] Marsden T., Sonnino R. (2008). Rural development and the regional state: Denying multifunctional agriculture in the UK. J. Rural Stud..

[6] Meyer E.L., Overen O.K., Obileke K., Botha J.J., Ngqeleni V.D. (2021). Financial and economic feasibility of bio-digesters for rural residential demand-side management and sustainable development. Energy Rep..

[7] Dale A., Newman L. (2009). Social capital: A necessary and sufficient condition for sustainable community development?. Community Dev. J..

[8] Bourdieu P. (1986). The forms of capital (English version). Handbook of Theory and Research for the Sociology of Education.

[9] Coleman J.S. (1988). Social Capital in the Creation of Human Capital. Am. J. Sociol..

[10] Oberle M. (2016). Robert D. Putnam: Bowling Alone. The Collapse and Revival of American Community, New York: Simon and Schuster 2000, 541 S.

[11] Tuna E., Karantininis K. (2021). Agricultural cooperatives as social capital hubs – A case in a post-socialist country. JCOM.

[12] Engbers T.A., Thompson M.F., Slaper T.F. (2017). Theory and Measurement in Social Capital Research. Soc. Indic. Res..

[13] Costa D.L., Kahn M.E. (2003). Civic Engagement and Community Heterogeneity: An Economist’s Perspective. Perspect. Politics.

[14] Gui B., Sugden R. (2005). Economics and Social Interaction: Accounting for Interpersonal Relations.

[15] Bjørnskov C. (2008). Social Capital and Happiness in the United States. Appl. Res. Qual. Life.

[16] Bjornskov C. (2010). The Happy Few: Cross–Country Evidence on Social Capital and Life Satisfaction. Kyklos.

[17] Bruni L., Stanca L. (2008). Watching alone: Relational goods, television and happiness. J. Econ. Behav. Organ..

[18] Clark W., Yi D., Huang Y. (2019). Subjective well-being in China’s changing society. Proc. Natl. Acad. Sci. USA.

[19] Helliwell J.F., Putnam R.D. (2004). The social context of well–being. Philos. Trans. R. Soc. B Biol. Sci..

[20] Neira I., Bruna F., Portela M., García-Aracil A. (2018). Individual Well-Being, Geographical Heterogeneity and Social Capital. J. Happiness Stud..

[21] Zhang R.J. (2020). Social trust and satisfaction with life: A cross-lagged panel analysis based on representative samples from 18 societies. Soc. Sci. Med..

[22] Ateca-Amestoy V., Aguilar A.C., Moro-Egido A.I. (2014). Social Interactions and Life Satisfaction: Evidence from Latin America. J. Happiness Stud..

[23] Gleibs I.H., Morton T.A., Rabinovich A., Haslam S.A., Helliwell J.F. (2013). Unpacking the hedonic paradox: A dynamic analysis of the relationships between financial capital, social capital and life satisfaction. Br. J. Soc. Psychol..

[24] Kim B.J., Linton K.F., Lum W. (2015). Social capital and life satisfaction among Chinese and Korean elderly immigrants. J. Soc. Work.

[25] Li W., Lin H., Jin Z. (2019). Social Capital Availability: The Case of Farmers and Herdsmen from Inner Mongolia (in Chinese). Econ. Res. J..

[26] Roest K.D., Ferrari P., Knickel K. (2018). Specialisation and economies of scale or diversification and economies of scope? Assessing different agricultural development pathways. J. Rural Stud..

[27] Ge D., Long H., Qiao W., Sun D., Yang R. (2020). Effects of rural–urban migration on agricultural transformation: A case of Yucheng City, China. J. Rural Stud..

[28] Ma L., Long H., Tu S., Zhang Y., Zheng Y. (2020). Farmland transition in China and its policy implications. Land Use Policy.

[29] Jin J., He R., Gong H., Xu X., He C. (2017). Farmers’ Risk Preferences in Rural China: Measurements and Determinants. Int. J. Environ. Res. Public Health.

[30] Zhu Z., Ma W., Leng C., Nie P. (2020). The Relationship Between Happiness and Consumption Expenditure: Evidence from Rural China. Appl. Res. Qual. Life.

[31] Vroome T., Hooghe M. (2014). Life Satisfaction among Ethnic Minorities in the Netherlands: Immigration Experience or Adverse Living Conditions?. J. Happiness Stud..

[32] Li S., An P.L., Pan Z.H., Wang F.T., Li X.M., Liu Y. (2015). Farmers’ initiative on adaptation to climate change in the Northern Agro-pastoral Ecotone. Int. J. Disaster Risk Reduct..

[33] Lin H., Li W., Hou S. (2019). The Social Capitaland Life Satisfaction of Farmers and Herdsmen: Survey of Farmers and Herdsmen from Inner Mongolia (in Chinese). Issues Agric. Econ..

[34] Putnam R.D. (1997). The Prosperous Community: Social Capital and Public Life. Am. Prospect.

[35] Ostrom E., Walker J., Gardner R. (1992). Covenants with and without a sword: Self-governance is possible. Am. Political Sci. Rev..

[36] Wouter S., Souren A., Tom E. (2014). Social capital of entrepreneurs and small firm performance: A meta-analysis of contextual and methodological moderators. J. Bus. Ventur..

[37] Acquaah M., Amoako-Gyampah K., Nyathi N.Q. (2014). Measuring and Valuing Social Capital: A Systematic Review.

[38] Chell E. (2016). Social enterprise and entrepreneurship: Towards a convergent theory of the entrepreneurial process. Int. Small Bus. J..

[39] Li C.R., Lin C.J., Huang H.C. (2014). Top management team social capital, exploration-based innovation, and exploitation-based innovation in SMEs. Technol. Anal. Strateg. Manag..

[40] Lindstrand A., Hånell S.M. (2017). International and market-specific social capital effects on international opportunity exploitation in the internationalization process. J. World Bus..

[41] Gericke D., Burmeister A., L?We J., Deller J., Pundt L. (2017). How do refugees use their social capital for successful labor market integration? An exploratory analysis in Germany. J. Vocat. Behav..

[42] Li P., Tang L., Jaggi B. (2018). Social Capital and the Municipal Bond Market. J. Bus. Ethics.

[43] Garrett R.D., Gardner T.A., Fonseca T., Marchand S., Barlow J., de Blas D.E., Ferreira J., Lees A.C., Parry L. (2017). Explaining the persistence of low income and environmentally degrading land uses in the Brazilian Amazon. Post-Print hal-01682674, HAL.

[44] Scoppa V., Ponzo M. (2008). An Empirical Study of Happiness in Italy. BE J. Econ. Anal. Policy.

[45] Rodriguezpose A., Von Berlepsch V. (2014). Social Capital and Individual Happiness in Europe. J. Happiness Stud..

[46] Bartolini S., Bilancini E., Pugno M. (2013). Did the Decline in Social Connections Depress Americans’ Happiness?. Soc. Indic. Res..

[47] Howell R.T., Howell C.J. (2008). The Relation of Economic Status to Subjective Well-Being in Developing Countries: A Meta-Analysis. Psychol. Bull..

[48] Elgar F.J., Davis C.G., Wohl M.J., Trites S.J., Martin M.S. (2011). Social Capital, Health and Life Satisfaction in 50 Countries. Health Place.

[49] Bartus T. (2005). Estimation of marginal effects using margeff. Stata J..

[50] Bian Y., Logan J.R. (1996). Market transition and the persistence of power: The changing stratification system in urban China. Am. Sociol. Rev..

[51] Fukushima S., Uchida Y., Takemura K. (2021). Do you feel happy when other members look happy? Moderating effect of community-level social capital on interconnection of happiness. Int. J. Psychol..

[52] Leung A., Kier C., Fung T., Fung L., Sproule R. (2011). Searching for Happiness: The Importance of Social Capital. J. Happiness Stud..

[53] Abdala R.G., Binotto E., Borges J.A.R. (2022). Family farm succession: Evidence from absorptive capacity, social capital, and socioeconomic aspects. Rev. Econ. Sociol. Rural.

[54] Arampatzi E., Burger M.J., Novik N. (2016). Social Network Sites, Individual Social Capital and Happiness. J. Happiness Stud..

[55] Wang R., Xue D., Liu Y., Liu P., Chen H. (2018). The Relationship between Air Pollution and Depression in China: Is Neighbourhood Social Capital Protective?. Int. J. Environ. Res. Public Health.

[56] Kuss D.J., Griffiths M.D. (2011). Online Social Networking and Addiction—A Review of the Psychological Literature. Int. J. Environ. Res. Public Health.

[57] Kemperman A., Berg P., Weijs-Perrée M., Uijtdewillegen K. (2019). Loneliness of Older Adults: Social Network and the Living Environment. Int. J. Environ. Res. Public Health.

[58] Frey B.S., Stutzer A. (2002). What can Economists Learn from Happiness Research?. J. Econ. Lit..

[59] Hu L., Liu R., Zhang W., Zhang T. (2020). The Effects of Epistemic Trust and Social Trust on Public Acceptance of Genetically Modified Food: An Empirical Study from China. Int. J. Environ. Res. Public Health.

[60] Novotný J., Kolomazníková J., Humňalová H. (2017). The Role of Perceived Social Norms in Rural Sanitation: An Explorative Study from Infrastructure-Restricted Settings of South Ethiopia. Int. J. Environ. Res. Public Health.

[61] Tilman D., Cassman K.G., Matson P.A., Naylor R., Polasky S. (2002). Agricultural sustainability and intensive production practices. Nature.

[62] Lins K.V., Servaes H., Tamayo A. (2017). Social Capital, Trust, and Firm Performance: The Value of Corporate Social Responsibility during the Financial Crisis. J. Financ..

[63] Kawachi I., Berkman L.F. (2001). Social ties and mental health. J. Urban Health.

[64] Pfeil U., Arjan R., Zaphiris P. (2009). Age differences in online social networking—A study of user profiles and the social capital divide among teenagers and older users in MySpace. Comput. Hum. Behav..

[65] Jiang N., Renema J. (2021). Immigrant-Native Disparities in Happiness among Middle-Aged and Older Adults in Western European Countries: The Moderating Role of Social Capital. J. Aging Health.

[66] Huang J., Fang Y. (2021). Income Inequality, Neighbourhood Social Capital and Subjective Well-Being in China: Exploration of a Moderating Effect. Int. J. Environ. Res. Public Health.

